# The Art of Bioimmunogenomics (BIGs) 5.0 in CAR-T Cell Therapy for Lymphoma Management

**DOI:** 10.34172/apb.2024.034

**Published:** 2024-03-10

**Authors:** Dito Anurogo, Dewi Luthfiana, Nuralfin Anripa, Apriliani Ismi Fauziah, Maratu Soleha, Laila Rahmah, Hana Ratnawati, Teresa Liliana Wargasetia, Sari Eka Pratiwi, Riswal Nafi Siregar, Ratis Nour Sholichah, Muhammad Sobri Maulana, Taruna Ikrar, Yu Hsiang Chang, Jiantai Timothy Qiu

**Affiliations:** ^1^International Ph.D. Program in Cell Therapy and Regenerative Medicine, College of Medicine, Taipei Medical University, Taipei, 110301, Taiwan.; ^2^Faculty of Medicine and Health Sciences, Muhammadiyah University of Makassar, Makassar, South Sulawesi, 90221, Indonesia; ^3^Bioinformatics Research Center, Indonesian Institute of Bioinformatics (INBIO), Malang, East Java, 65162, Indonesia.; ^4^Department of Environmental Science, Dumoga University, Kotamobagu, South Sulawesi, 95711, Indonesia.; ^5^Faculty of Medicine Ramathibodi Hospital, Mahidol University, Bangkok, 10400, Thailand.; ^6^MSc Program in Tropical Medicine, Kaohsiung Medical University, Kaohsiung City, 807378, Taiwan.; ^7^National Research and Innovation Agency (BRIN), Central Jakarta, 10340, Indonesia.; ^8^IKIFA College of Health Sciences, East Jakarta, Special Capital Region of Jakarta, 13470, Indonesia.; ^9^Department of Digital Health, School of Medicine, Tehran University of Medical Sciences, Tehran, 1416634793, Iran.; ^10^Faculty of Medicine, Muhammadiyah University of Surabaya, Surabaya, East Java, 60113, Indonesia.; ^11^Faculty of Medicine, Maranatha Christian University, Bandung, West Java, 40164, Indonesia.; ^12^Department of Biology and Pathobiology, Faculty of Medicine, Tanjungpura University, Pontianak, West Kalimantan, 78115, Indonesia.; ^13^Department of Biotechnology, Postgraduate School of Gadjah Mada University, Yogyakarta, 55284, Indonesia.; ^14^Community Health Center (Puskesmas) Temon 1, Kulon Progo, Special Region of Yogyakarta, 55654, Indonesia.; ^15^Director of Members-at-Large, International Association of Medical Regulatory Authorities (IAMRA), Texas, 76039, USA.; ^16^Aivita Biomedical Inc., Irvine, California, 92612, USA.; ^17^Chairman of Medical Council, The Indonesian Medical Council (KKI), Central Jakarta, 10350, Indonesia.; ^18^Adjunct Professor, School of Military Medicine, The Republic of Indonesia Defense University (RIDU), Jakarta Pusat, 10440, Indonesia.; ^19^Department of Pharmacology, Faculty of Medicine, Malahayati University, Bandar Lampung, Lampung, 35152, Indonesia.; ^20^Locus Cell Co., LTD., Xizhi Dist., New Taipei City, 221, Taiwan.; ^21^Department of Obstetrics and Gynecology, School of Medicine, College of Medicine, Taipei Medical University, Taipei, 110301, Taiwan.; ^22^Department of Obstetrics and Gynecology, Taipei Medical University Hospital, Taipei, 110301, Taiwan.

**Keywords:** CAR-T Cells, Lymphoma, Management

## Abstract

**Purpose:**

Lymphoma, the most predominant neoplastic disorder, is divided into Hodgkin and Non-Hodgkin Lymphoma classifications. Immunotherapeutic modalities have emerged as essential methodologies in combating lymphoid malignancies. Chimeric Antigen Receptor (CAR) T cells exhibit promising responses in chemotherapy-resistant B-cell non-Hodgkin lymphoma cases.

**Methods:**

This comprehensive review delineates the advancement of CAR-T cell therapy as an immunotherapeutic instrument, the selection of lymphoma antigens for CAR-T cell targeting, and the conceptualization, synthesis, and deployment of CAR-T cells. Furthermore, it encompasses the advantages and disadvantages of CAR-T cell therapy and the prospective horizons of CAR-T cells from a computational research perspective. In order to improve the design and functionality of artificial CARs, there is a need for TCR recognition investigation, followed by the implementation of a quality surveillance methodology.

**Results:**

Various lymphoma antigens are amenable to CAR-T cell targeting, such as CD19, CD20, CD22, CD30, the kappa light chain, and ROR1. A notable merit of CAR-T cell therapy is the augmentation of the immune system’s capacity to generate tumoricidal activity in patients exhibiting chemotherapy-resistant lymphoma. Nevertheless, it also introduces manufacturing impediments that are laborious, technologically demanding, and financially burdensome. Physical, physicochemical, and physiological limitations further exacerbate the challenge of treating solid neoplasms with CAR-T cells.

**Conclusion:**

While the efficacy and safety of CAR-T cell immunotherapy remain subjects of fervent investigation, the promise of this cutting-edge technology offers valuable insights for the future evolution of lymphoma treatment management approaches. Moreover, CAR-T cell therapies potentially benefit patients, motivating regulatory bodies to foster international collaboration.

## Introduction

 Lymphomas, which represent a predominant class of lymphoid neoplasms, constitute a diverse group of malignancies resulting from the clonal expansion of lymphocytes.^1,2^ These cancers are classified into two primary types: Hodgkin’s lymphoma (HL) and non-Hodgkin’s lymphoma (NHL). Hodgkin’s lymphoma is further subdivided into non-classical Hodgkin’s lymphoma and classic Hodgkin’s lymphoma.^1,3,4^ NHL includes numerous subtypes, with the most prevalent being diffuse large B-cell lymphoma (DLBCL) (25-30%), accounting for over 30% of B-NHL cases.^5-8^ In contrast to Hodgkin’s lymphoma, 90% of cases belong to the NHL category, which includes B-cell NHL (B-NHL) expressing markers CD20, CD19, or CD22, T-cell NHL (T-NHL) expressing CD3, CD4, or CD8, and natural killer (NK)/T cell NHL expressing CD56.^9-11^

 The lymphatic system, an integral component of the immune system,^12^ is crucial in combating infections and disease while facilitating fluid transport throughout the body.^13^ B and T lymphocytes residing in lymphatic tissue contribute to defense against infectious agents. Chronic antigenic stimulation increases B and T cell proliferation, raising the risk of genetic anomalies and triggering lymphoma.^6^ Lymphomas can originate in any region containing lymphatic tissue, including the spleen,^14^ bone marrow,^9^ thymus,^15^ tonsils,^16^ and lymph nodes.^17^

 As of 2021, lymphoma is estimated to have affected 825,651 individuals in the United States.^18^ In 2022, it is estimated that there will be 91 010 new cases (82 470 NHL cases and 8540 HL cases) with a predicted death toll of 170 people,^19^ accounting for 3.5% of global cancer fatalities.^20,21^ While lymphoma-associated mortality rates remain low, lymphoma risks developing secondary malignancies, such as lung, kidney, breast, colorectal, and skin melanoma, if inadequately treated.^19,22^ Similar to breast cancer, radiotherapy increases the cancer susceptibility.^23^ T cell dysfunction in HL and DLBCL has been linked to an elevated risk of skin melanoma.^24^ Additionally, a family history of lung, colorectal, or breast cancer further increases the likelihood of secondary malignancies,^19^ potentially leading to higher mortality rates if lymphoma triggers a secondary cancer.

 Tumor development exhibits variability across stages; however, tumors generally display cellular infiltration resembling centrocytes. Lymphomas are characterized by small size, abundant cytoplasm, and an irregular nucleus. Lymphoma cells express immunoglobulins IgM, IgG, and IgA. Subsequently, NHL exhibits increased expression of CD19, CD20, and CD22 receptors.^6^ In contrast, HL predominantly expresses the characteristic immunophenotypic pattern of CD15, CD30, and CD45 receptors.^4^

## Immunotherapeutic approaches and CAR-T cell therapy for lymphoma

 Immunotherapy, a therapeutic approach that aims to enhance the functionality of immune cells to eliminate neoplastic cells, has made significant advancements in oncological treatment and yielded clinically meaningful outcomes. Various immunotherapy approaches have rapidly developed, including oncolytic virus therapy, tumor-specific antigens as cancer vaccines, dendritic cell-based cancer vaccination, genetic modification of autologous tumor cells to evoke tumor-specific immune responses, the application of cytokines in cancer treatment, adoptive cell transfer (ACT), and immune checkpoint inhibitors. Advances in genetic engineering have led to numerous innovations in cancer immunotherapy, emphasizing the importance of immunotherapy in clinical applications for cancer treatment. Immunotherapy primarily focuses on activating T cells due to their potent tumor-killing capability.^25^

 ACT employs autologous T cells, which undergo isolation, genetic engineering, and ex-vivo expansion before being reinfused into the patient to eliminate cancerous cells. Initially, ACT utilized tumor-infiltrating lymphocytes (TILs), but this approach induced persistent clonal repopulation of T cells in cancer patients. Consequently, ACT has evolved to incorporate genetically engineered T cells targeting specific neo-antigens, which are then reinfused into the patient for cancer cell eradication.^25,26^

 Two primary categories of genetically engineered T cells exist: T-cell receptor (TCR)-engineered T cells and chimeric antigen receptor (CAR)-T cells. The underlying principle of CAR-T cells involves the genetic engineering of TCRs with elevated tumor antigen avidity, known as CARs, which are subsequently cloned or ex-vivo expanded and transduced into patient T cells. This methodology directs cytotoxic T-cell activity toward tumor antigens.^27^ CAR-T cells have been primarily employed in treating hematological malignancies, including acute lymphoblastic leukemia, chronic lymphocytic leukemia, lymphoma, and multiple myeloma. The most efficacious CAR targets CD19, a crucial biomarker of the B cell lineage. Autologous T cells undergo genetic modification to target CD19 B-cell antigens via the expression of anti-CD19.^28^

 CAR-T cells represent one of the most cutting-edge immunotherapeutic approaches for relapsed or chemotherapy-refractory B-cell NHL, particularly for patients who have shown no resolution following multiple courses of chemotherapy.^29^

## Choice of lymphoma antigen for CAR-T targeting

**Figure 1:**
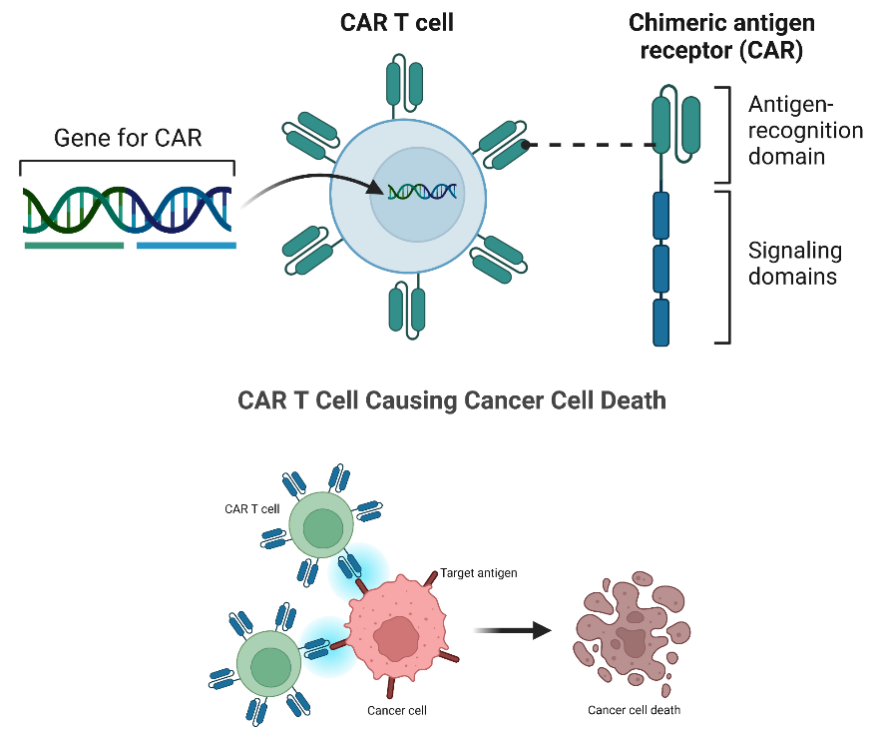
CAR-T cell structure and its role in targeting cancer cells(Edited in BioRender.com)

 Various therapies, from monoclonal antibodies to CAR-T cells, have been developed to treat lymphoid malignancies.^7,9,10^ CARs are composite constructs composed of multiple domains derived from various proteins, including (1) an antigen recognition domain typically sourced from an antibody, (2) a CD3 T-cell co-receptor signaling domain and (3) a co-stimulatory domain necessary for T-cell activation during antigen presentation (Figure 1).^30,31^

 CARs consist of four components: an extracellular domain, typically comprising single-chain variable fragments (scFv); a hinge region; a transmembrane domain; and an intracellular domain encompassing a T-cell activation domain and a co-stimulatory domain, both of which contribute to CAR-T cell activation and proliferation.^7,8,10^ CAR molecules target antigens on the surface of tumor cells, which may include compounds composed of carbohydrates, glycolipids, and proteins. The interaction between CAR and these targets creates immune synapses, resulting in contact-dependent cytotoxicity.^32^

 Modifications and amalgamations of CAR-T cells have been devised to enhance therapeutic effectiveness while minimizing adverse effects. The CAR-T cell constructs that have been developed encompass armored CAR, tandem CAR, multi-CAR, and switchable CAR.^33^ The appropriate antigen for lymphoma therapy with CAR-T cells should be selected following the molecular markers of both B and T cell lymphoma subtypes.^10^

 CD19, a transmembrane glycoprotein regulating B cell activation in an antigen receptor-dependent manner, is an optimal target for CAR-T therapy of B-cell lymphoma, as CD19 is expressed throughout B cell differentiation and frequently during B cell malignant transformation. Furthermore, CD19 is a ubiquitously expressed antigen on the surface of B cells, including B-NHL.^7,8,11^ The most widespread and popular clinical treatment strategy is CAR-T cell therapy targeting CD19.^34^ In relapsed/refractory (R/R) aggressive B-cell NHLs, the CD19 marker in malignant cells is lost, necessitating alternative antigens for targeting these malignant cells. CAR-T cells targeting CD20 or CD22 under R/R conditions have been investigated, albeit in preliminary stages.^10,11,35,36^ Other potential targets for various lymphoma types include CD30,^37^ k-light chain (Table 1),^38^ and receptor-tyrosine-kinase-like orphan receptor 1 (ROR1).

**Table 1:** Choice of lymphoma antigen for CAR-T cell targeting

**CAR Configuration**	**Target Antigen**	**Co-stimulatory Domain**	**Pathology/Disease**	**Outcome**	**Ref.**
CD19-4-1BB-CD3ζ	CD19	4-1BB	B Cell Lymphoma	CR: 3/20 PR: 1/20	^41^
CD19-CD28-CD3ζ	CD19	CD28		CR: 1/8; PR: 5/8; SD: 1/8	^42,43^
CD19-CD28-CD3ζ	CD19	CD28		CR: 8/15; PR: 4/15; SD: 1/15	^44^
CD19-CD28-CD3ζ	CD19	CD28		CR: 1/9; PR: 5/9	^45^
CD20-CD3ζ	CD20	None		CR: 2/7; PR: 1/7; SD: 4/7	^46^
CD19-CD3ζ	CD19	None		CR: 7/16; PR: 4/16	^47^
CD20-4-1BB-CD3ζ	CD20	4-1BB		CR: 3/6; PR: 1/6	^48^
k-Light Chain- CD3ζ	k-light chain	None		CR: 2/10; PR: 3/10	^38^
CD30-4-1BB-CD3ζ	CD19/CD30	4-1BB	HL/ALCL	CR: 1/6; PR: 3/6; PD: 2/6	^49^
CD19-4-1BB-CD3ζ	CD19	4-1BB (CD137)	Lymphoma	-	^28^
CD19-CD28-CD3ζ	CD19	CD28			
CD19-CD28-CD3ζ	CD19	CD28			
CD19-CD28-CD3ζ	CD19	CD28			
CD19-CD28-CD3ζ	CD19	CD28			
CD19-CD28-4-1BB-CD3ζ	CD19	CD28 and 4-1BB			
CD19-CD28-CD3ζ	CD19	CD28	Acute Non-Chronic Lymphoma	CR: 6/20, PR: 2/20	^50,51^
CD19-CD28-CD3ζ	CD19	CD28	B Cell Lymphoma	CR: 3/8, PR: 1/8	^51,52^
CD19-CD28-CD3ζ	CD19	CD28	Acute and Chronic Lymphoma	CR: 11/19	^51,53^
CD19-4-1BB-CD3ζ	CD19	4-1BB	Acute Lymphoma	CR:1/2	^34,51^
CD19-CD28/CD137/CD27-CD3ζ	CD19	CD28/CD137/CD27	Acute Lymphoma	CR:4/6	^51,54^
CD19/CD20-CD3ζ	CD19/CD20	None	FL/DLBCL	RR: 2/4, CR: 2/4	^35^
CD19-CD28-CD3ζ	CD19	CD28	SLL/TFL/PCNSL/ DLBCL	RR: 0/6, CR: 0/6	^55^
CD19-CD28-CD3ζ	CD19	CD28	FL/SMZL	RR: 4/5, CR: 0/5	^43^
CD20-CD28-4-1BB-CD3ζ	CD20	CD28 and 4-1BB	MCL/FL	RR: 3/3, CR: 2/3	^56^
CD20-4-1BB-CD3ζ	CD20	4-1BB	DLBCL	RR: 5/7, CR: 1/7	^48^
CD19-CD28-CD3ζ	CD19	CD28	SMZL/PMBCL/DLBCL/low-grade NHL	RR: 8/11, CR: 5/11	^44^
CD19-CD28-CD3ζ	CD19	CD28	DLBCL/MCL/TFL	RR: 2/10, CR: 1/10	^50^
k-Light Chain-CD28-CD3ζ	k-Light Chain	CD28	DLBCL/MCL/TFL/LPL	RR: 3/7, CR: 2/7	^38^
CD19-CD28-CD3ζ	CD19	CD28 and 4-1BB	LBCL/TFL.MCL/FL	RR: 19/30, CR: 10/30	^47^
CD20-CD28-CD3ζ/ CD19-CD3ζ	CD19	None/ CD28	DLBCL/MCL	RR: 15/16, CR: 13/16	^57^
CD20-4-1BB-CD3ζ	CD20	4-1BB	DLBCL/FL/MCL/PCMZL	RR: 9/11, CR: 6/11	^58^
CD19-CD28-CD3ζ	CD19	CD28	DLBCL/FL/PMBCL/MCL	RR: 16/22, CR: 12/22	^45^
CD19-CD28-CD3ζ	CD19	CD28	DLBCL	RR: 5/7, CR: 4/7	^59^
CD19-CD28-CD3ζ	CD19	CD28	DLBCL/FL/PMBCL	RR: 83/101, CR: 55/101	^60^
CD19-CD28-CD3ζ	CD19	CD28	DLBCL/FL	RR: 18/28, CR: 16/28	^29^
CD19-CD28-4-1BB-CD3ζ	CD19	CD28 and 4-1BB	DLBCL/MCL/FL	RR: 3/9, CR: 3/9	^61^
CD19-CD28-4-1BB-CD3ζ	CD19	CD28 and 4-1BB	DLBCL/SLL/BCLU	RR: 9/13, CR: 7/13	^62^

 ROR1, one of the most promising cancer targets, is aberrantly expressed in numerous malignancies yet exhibits minimal expression in normal tissue, rendering it a viable candidate for CAR-T therapy.^39^ ROR1 may represent a tumor-specific target for treatment since it was found to be significantly expressed in B-cell chronic lymphocytic leukemia (B-CLL) but not in normal B cells.^40^ ROR1-targeted T-cell therapies could be advantageous in treating B-CLL and other ROR1-positive cancers.

 Multiple clinical trials have discovered that CAR-T cells with specific targets exhibit a broad range of therapeutic effects in various lymphoma types, as demonstrated in Table 1, such as Hodgkin lymphoma (CD19/CD30); Anaplastic large cell lymphoma (CD30); Follicular lymphoma (CD19/CD20); Diffuse large B-cell lymphoma (CD19/CD20/k-light chain); Small lymphocytic lymphoma (CD19); Transformed follicular lymphoma (CD19/ k-light chain); Primary central nervous system lymphoma (CD19); Splenic marginal lymphoma (CD19); Mantle cell lymphoma (CD19/CD20/k-light chain); Primary mediastinal large B cell lymphoma (CD19); NHL (CD19); Lymphocyte lymphoma (k-light chain); Large B cell lymphoma (CD19); Primary skin marginal zone B-cell lymphoma (CD19/CD20); B-cell lymphoma characterized by DLBCL and Burkitt lymphoma (CD19). These patients received treatment with co-stimulatory molecules, including CD28, 4-1BB + CD28, 4-1BB (CD137), or CD37.

## Design, manufacturing, delivery, and global regulatory aspects of successful CAR-T cell therapy

###  Design of CAR-T cells

 Recent studies on TCR recognition have the potential to improve the design and function of synthetic CARs. Factors such as K_on_/K_off_ rates, spatial constraints between T-cells and antigen-presenting cells (APCs), immunological synapse formation, TCR clustering, and interactions of CD4 and CD8 co-receptors with major histocompatibility complex (MHC)/peptide complexes determine TCR affinity for MHC/peptide complexes, affecting downstream signaling, T cell function, and cell fate.^63^

 Active APCs play a role in co-stimulating TCR signaling. Some organizations are developing tumor-specific CARs that aim not to identify normal cells by co-expressing two CARs with separate binding domains.^64^ However, this dual receptor approach involving CARs and antigens presents challenges. For instance, Wilkie et al. targeted overexpressed Her2 and Muc1 in cancers using a split receptor approach. Muc1-specific CARs had CD28 co-stimulatory domains, while Her2-binding CARs had CD3 signaling domains,^65^ resembling T cell signal 1 and 2 checkpoints. Dual-specific CAR-T cells were only generated when Her2/Muc1 double-positive target cells delivered signals 1 and 2, while single-positive target cells did not develop them.^66^ Dual-specific CAR-T cells successfully destroyed Her2 + Muc1 + /- cells in vitro. Grada et al. encountered similar issues with their split receptor system, as dual-specific CAR-T cells killed CD19 + and Her2 + target cells despite targeting only one type.^67^ Since they cannot discriminate between single-positive and double-positive target cells, these CARs cannot enhance the specificity of single-input CAR-T targeting specificity. Kloss et al. demonstrated that “signal 1” CARs can be switched off, leaving only the co-stimulatory CAR-T cells to activate T cells fully. They tested PSCA-targeting CAR-scFvs.^68^ The poorly produced CARs by T cells failed to eliminate PSCA + tumor cells in mice or the lab. However, an alternative approach involving a less-than-ideal CAR and a second co-stimulatory CAR that conveyed signals for CD28 and 4-1BB allowed T cells to recognize both PSCA + and PSMA + tumor cells in vivo. This technique targets CARs and reduces non-tumor effects.^69^

 CARs achieve tumor specificity through a divided receptor system that signals negatively to normal tissue but not tumor tissue. In conventional T-cell biology, dominant TCR signaling suppression is advised.^70^ T-cells activate PD-1/CTLA-4, which disrupts TCR signaling either by competing for co-stimulatory ligands (such as CTLA-4 for CD28-ligands CD80 and CD86) or by binding to APC ligands activated by pro-inflammatory cytokines (e.g., PD-1 for PD-L1 and PD-L2).^71^ Negative feedback mechanisms reduce pro-inflammatory T-cell activation and host harm. Mice deficient in PD-1 and CTLA-4 develop autoimmune and lymphoproliferative diseases. PD-L1 is produced by ocular neurons and placental trophoblasts, contributing to immune-protected tissues.^72^ In chronic infections and cancers, “tired” T cells with prolonged antigen exposure activate inhibitory receptors. In clinical settings, PD-1 and CTLA-4 immunotherapies activate T cells to viral and tumor antigens.^73^

 Modulating CAR recognition through the co-expression of a second inhibitory CAR specific for an antigen produced in average but not malignant tissue allows CAR-T cells to avoid normal tissue. Fedorov et al. demonstrated that co-expressing a CD19-specific activating CAR with CD3 and CD28 endodomains and a PSMA-specific inhibitory CAR with PD-1 or CTLA-4 endodomains was effective.^74^ CAR-T cells could stimulate CD19-expressing targets, while PSMA-expressing target cells could not multiply, kill target cells, or release pro-inflammatory cytokines. Enhancing CAR activation selectivity for ligands produced on some normal and some malignant cells is intriguing.^75^ However, creating inhibitory CARs is challenging due to a limited understanding of TCR signaling inhibitory receptors. Another challenge is selecting ligands binding exclusively to normal cells to transmit a negative signal. PD-1 blocks TCR signaling by co-localizing with TCR microclusters at the synapse,^76^ attracting SHP2 and dephosphorylating proximal TCR signaling proteins, such as CD3, Zap70, and PKC. The PD-1 endodomain may require the inhibitory CAR-T close to the activating CAR. CTLA-4 competes with CD28 for binding to CD80/CD86 and physically obstructs CD28 from the synapse, reducing co-stimulatory and TCR signaling.^77^ Recent research suggests that the CTLA-4 endodomain modulates surface CTLA-4 expression without inhibitory signals. Co-stimulatory endo-domains in second- and third-generation activating CARs can reduce their efficacy, with PD-1 potentially better at suppressing second-generation CAR activity than CTLA-4.^78^

 Improved assays are needed to understand better how split receptors differ from TCR signaling and how they might be modified to mimic it better. These assays should track proteins during immune synapse development using planar lipid bilayers as fake APCs. This method can determine how many ligands activate or inhibit co-expressed CARs and whether co-stimulatory or co-inhibitory CARs target highly expressed antigens. Studies indicate that co-stimulatory or co-inhibitory receptors must co-localize with TCRs at the synapse to stimulate or delay the immune response. Confocal microscopy should provide insights into CAR signaling in terms of location and time and how CAR split receptor systems differ from T cell signaling.^79,80^

###  Production of CAR-T cells

 Quality control is essential during CAR-T cell manufacturing. Leukapheresis is the process of extracting leukocytes from the patient’s blood and returning it to circulation. This procedure enriches T cells. Leukapheresis is followed by washing the cells in an anticoagulant-containing buffer. Counterflow centrifugal elutriation separates cells based on size and density, further enhancing the enrichment of lymphocytes. The next step involves separating T cells into CD4 and CD8 subsets using antibody bead conjugates or markers.^81,82^

 The activation of T cells with patient APCs involves multiple steps. Achieving a potent CAR-T cell product is a complex and time-consuming process. Beads coated with anti-CD3/anti-CD28 monoclonal antibodies have proven more reliable and effective for stimulating T cells (Life Technologies). While IL-2, feeder cells, and anti-CD3 antibodies have been used for years, the activation and proliferation of T cells outside the body are less efficient compared to the use of beads coated with anti-CD3/anti-CD28 monoclonal antibodies or cell-based artificial antigen-presenting cells (aAPC).^83^ Magnetic separation simplifies the removal of aAPC beads during culture. Interleukin-2 and aAPC can support the logarithmic growth of T cells in a perfusion bioreactor for weeks. K562 aAPC can produce co-stimulatory ligands and expand T lymphocytes ex vivo. Altering the culture conditions can bias T cells towards a Th2 or Th17 phenotype, and preclinical studies have shown good performance with Th17-polarized CAR-T cells. Clinical T-cell polarization may also be feasible.^84^

 CAR-containing viral vectors are used to stimulate T lymphocytes. After several days, the viral vectors are eliminated through dilution or media change. Viral machinery links the viral vectors to patient cells, providing them with RNA. CAR-T cell therapy generates CARs from genetic information, permanently altering the patient’s genetics by reversing RNA into DNA. Bioreactor cells continuously express CARs, displaying them on the cell surface. For CAR-T cell research, CTL019 employs retroviral vectors, known for having safer integration sites than gamma-retrovirals. T cell CAR expression has also been investigated using the Sleeping Beauty transposon system and mRNA transfection. Clinical use of transient mRNA-transfected CAR-T cells require multiple infusions. Despite its lower cost and early clinical trials, concerns persist about the efficacy of the Sleeping Beauty transposon system compared to lentiviral vectors, the unexplored potential of insertional mutagenesis, and the possibility of transposons “jumping” again.^85,86^

###  Delivery of CAR-T cells

 The treatment of solid tumors with CAR-T cells faces significant challenges due to physical, physicochemical, and physiological constraints. Physical and physicochemical barriers hinder the aggregation and growth of CAR-T cells within the tumor, while physiological obstacles, such as the unique cells within the tumor and the immune-suppressing properties of the tumor microenvironment (TME), enable the tumor to evade T cells and impede their function. Addressing these significant challenges is crucial to enhancing the effectiveness of CAR-T cell therapy for solid tumors.^87^ CAR-T cells can communicate with cell-loading devices close to solid tumors. These devices facilitate the development and movement of CAR-T cells. Innovative bioresorbable CAR-T cell-loading devices can sustain CAR-T cells’ viability and functionality within the tumor, eventually disintegrating within biological tissues.^88^ Although these cell-loading devices can offer long-term delivery of therapeutic cells, further research is needed to ensure patient safety and assess their impact on malignancies. Micro- and nano-devices capable of infiltrating solid tumors may assist CAR-T cells in overcoming the tumor stroma and improving their anti-cancer efficacy.^89^

 CAR-T cell therapy can complement other treatments for solid cancers. Combining two or more therapies can synergistically enhance therapeutic benefits and reduce adverse effects. Photothermal treatment, for instance, induces mild hyperthermia to facilitate the infiltration of CAR-T cells into solid tumors. In cases of deep tumors, non-invasive methods like focused ultrasound or radio-frequency electromagnetic radiation can induce hyperthermia.^90^ Cytokines and chemokines play a crucial role in immune responses, and improved CAR-T cells can release these immune-boosting molecules. Research on cytokines and chemokines can lead to the development of CAR-T cell immunomodulators. Another approach is the injection of tumor-specific cytokines through bioresponsive drug delivery. Solid tumor CAR-T cell therapy must aim to avoid cytokine release syndrome (CRS) and “on-target, off-tumor” side effects. Strategies for controlled activation and elimination of CAR-T cells are being explored, but clinical testing is required.^91,92^

 Furthermore, advancements in gene-editing technologies, such as CRISPR/Cas9, are poised to revolutionize CAR-T cell therapy. These tools can be used to engineer CAR-T cells, making them more resilient against the immunosuppressive tumor microenvironment, improving their tumor-targeting efficiency, and reducing off-tumor cytotoxicity. For example, removing immune checkpoint molecules like PD-1 in CAR-T cells may enhance their resistance to the suppressive effects induced by solid tumors.^93^ Additionally, the concept of ‘armored CAR-T cells,’ designed to secrete immune-stimulating cytokines or pro-inflammatory molecules, could bolster their anti-tumor potency by counterbalancing the immunosuppressive environment and recruiting other immune effector cells to the tumor site. Finally, to enhance the precision and safety of CAR-T cell therapy, researchers are exploring inducible CAR-T cell systems, allowing external modulation of CAR expression and activity. These cutting-edge approaches have the potential to toggle CAR-T cell activity “on” and “off” as needed, significantly enhancing the safety profile of this powerful therapeutic intervention. Future research and clinical efforts will determine the optimal strategies for incorporating these novel advances into the next generation of CAR-T cell therapies for solid tumors.

###  Global regulatory considerations for successful CAR-T cell therapy

 CAR-T cell therapies hold great promise, and their widespread success hinges on harmonizing global regulations. These therapies offer significant benefits to patients, which may prompt regulatory agencies worldwide to collaborate and enhance international cooperation. The regulation of this emerging therapy is becoming a global challenge.^93^ The FDA has long played a crucial role in regulating and providing guidance on cell and gene therapy. However, the regulatory landscape varies among major countries, each with its approach to evaluating clinical trial submissions. For example, the EU requires a qualified person to assess compliance with current good manufacturing practices (cGMP), while the US focuses on document review. As more countries participate in clinical trials, the manufacturing process must become more robust to address different regulatory authorities’ diverse criteria and questions.^94^ Efforts to harmonize global regulations for CAR-T cell therapies began in earnest when, on October 11, 2012, nine members of the global regulatory community, including ANVISA, Brazil’s European Medicines Agency (EMA), Health Canada, India’s National Institute of Biologicals (NIB), Japan’s Ministry of Health, Welfare, and Labor/Pharmaceutical and Medical Devices Agency, South Korea’s Ministry of Food and Drug Safety (formerly KFDA), and Singapore’s Health Sciences Authority, met to facilitate collaboration among developers of cell and gene therapies.^95,96^ Product development in this field remains unpredictable, and regulators often rely on their judgment, lacking unified norms and cross-domain experience. Each country’s unique production requirements further complicate matters, encompassing nationwide screening, testing, tracing, labeling, patient privacy, and apheresis standards. The international distribution of donor materials and finished products complicates the regulatory landscape. Definitions and quality control standards for starting materials vary by region.^97^ Multi-country testing should balance accommodating individual country requirements and maintaining the quality of starting materials. It is essential to ensure the accessibility of information related to reagent origin, provenance, content, and certification. Furthermore, it is worth noting that each region has its guidelines for materials used in cell and gene therapies, including those related to cell culture serum.^98^ To address these regulatory challenges, efforts are underway to develop methods for producing CAR-T cells using chemicals that meet quality criteria accepted in multiple regions. It includes, for example, adopting the same international supplier across global regions.^99^

 Achieving widespread success with CAR-T cell therapies necessitates a harmonized approach to clinical trial regulations. It entails reaching a consensus among regulatory authorities on the design of clinical trials, endpoints, and the targeted patient population. With varying definitions of clinical efficacy across countries, especially in multi-country trials, there is a growing need for globally accepted, uniform clinical efficacy and safety standards. Additionally, harmonized guidelines for managing and reporting adverse events are crucial, given the unique and potentially severe side effects associated with CAR-T cell therapy. Regulatory bodies must agree on toxicity grading scales and management strategies to ensure the well-being of patients across diverse healthcare systems.

 Furthermore, requirements for long-term patient follow-up following CAR-T cell therapy may vary between countries, highlighting the importance of a global consensus on the appropriate duration and scope of these follow-ups. It not only aids in monitoring late-onset adverse events but also assesses the therapy’s long-term efficacy. Finally, given the rapid technological advancements, regulatory frameworks must exhibit flexibility and adaptability to accommodate the distinctive characteristics of emerging CAR-T and other cell and gene therapies.

###  Advantages, drawbacks, and challenges of CAR-T cell therapy 

 CAR T-cell therapy is the subject of extensive investigation due to its potential in treating specific malignancies, particularly leukemias and lymphomas.^100^ This therapy offers several advantages but presents drawbacks and challenges that must be addressed.

 One significant advantage of CAR-T cell therapy is its ability to enhance the immune system’s tumor-killing activity in patients with chemotherapy-resistant lymphoma.^8^ It has shown promising results in improving the prognosis of individuals with certain types of low-risk lymphoma.^101^ Additionally, anti-CD19 CAR-T cell therapy has demonstrated remarkable effectiveness in relapsed or refractory aggressive B-cell lymphomas.^102^

 However, there are certain drawbacks associated with CAR-T cell therapy. Disease progression or recurrence is a common concern among patients undergoing this treatment.^101^ Challenges arise during the manufacturing process, where insufficient harvesting of CAR-T cell products or inadequate expansion of generated CAR-T cells can occur. Immediate recurrence is observed in many patients due to poor CAR-T cell persistence or cancer cell resistance resulting from antigen loss or modulation, such as the loss or downregulation of CD19 and/or CD22 on malignant B cells.^103^

 Two common toxicities associated with CAR-T cell therapy are CRS and immune effector cell-associated neurotoxicity syndrome (ICANS). Current strategies for managing these toxicities involve careful monitoring, early detection, and timely intervention with supportive care or administering tocilizumab and corticosteroids for severe cases.^8,102^ Technical and financial challenges are also encountered in manufacturing patient-specific batches of CAR-T cell products.^28^

 Several challenges lie ahead in the field of CAR-T cell therapy. There is a need to improve the therapy’s efficiency further and reduce response times.^8^ Identifying the subtypes of lymphoma that are most likely to benefit from CAR-T cell therapy is crucial.^101^ Developing patient-specific therapies^103^ and overcoming obstacles to treatment while enhancing efficacy and minimizing toxicity are essential objectives.^101^ Exploring prophylactic strategies to reduce toxicity without compromising efficacy is another area of focus.^102^ Multi-antigen-targeting strategies should be considered to address disease recurrence mechanisms and potentially achieve more durable remissions.^103^ Furthermore, determining the appropriate circumstances to choose CAR-T cell therapy over other promising modalities, such as bispecific antibodies, requires careful consideration.^101^

## Safety of CAR-T cells for lymphoma

 CAR-T cell therapy is associated with several safety concerns, including toxicity, cytokine release syndrome, graft-versus-host disease (GVHD), cytopenia (thrombocytopenia, anemia, and neutropenia), as well as febrile neutropenia and neurotoxicity. Among 16 studies evaluating the incidence of neurotoxicity during CAR-T therapy, a rate of 9% was reported. Neurotoxicity presents as a series of reversible neurological syndromes with unknown pathogenesis, and grade 3-4 neurotoxicity can be life-threatening.^104^ The etiology may be attributed to the absence of pre-conditioning prior to treatment.^105^ Importantly, no significant difference in neurotoxicity was observed between various cancer types.

 The most common adverse effect observed is CRS, characterized by forming a cytokine storm that can cause tissue damage. In severe cases, immediate intervention is necessary.^104^ CRS is the most frequently reported toxicity and manifests with symptoms such as high fever, rigors, sweating, anorexia, headache, myalgia/arthralgia, altered mental status, nausea/vomiting, and potential progression to life-threatening capillary leak with hypoxia, hypotension, and multiorgan dysfunction.^104,106,107^ Dramatic elevations of cytokines, including interferon-gamma, granulocyte-macrophage colony-stimulating factor, IL-10, and IL-6, have been observed following CAR-T cell infusion.^106^

 Additionally, acute and chronic GVHD after alloHCT were observed in 2 and 6 patients, respectively. Four patients showed evidence of graft-versus-leukemic activity following immunomodulation (tapering down immunosuppression, transfusion of donor lymphocytes, or administering checkpoint inhibitors). Importantly, when leukapheresis, no patient exhibited evidence of GVHD or immunocompromised CAR-T cell generation. Moreover, no patient showed GVHD or immunosuppression during leukapheresis for CAR-T cell production. Except for one CLL patient with 15% circulating tumor cells, all other evaluable patients demonstrated complete donor chimerism in peripheral blood during leukapheresis. All patients had active disease during lymphodepletion despite prior bridging therapy in 7 of 10 cases.^108^

## Future perspectives on CAR-T cell therapy in lymphoma management

 Successful implementation of CAR-T cell therapy requires better toxicity grading and management strategies to address the toxic side effects and other obstacles to its application.

 CAR-T treatment’s two frequent and potentially fatal side effects are CRS and neurotoxicity. Gaining a deeper understanding of these toxicities’ underlying mechanisms and causes will lead to improved toxicity management and increased CAR-T efficacy. Furthermore, it is essential to expand the benefits of CAR-T therapy to solid tumors promptly, as most clinical trials and studies to date have focused on liquid diseases like lymphomas.^109^

 More research is needed to identify response predictors and enhance the benefit-to-risk profile, ultimately reducing toxicity and treatment burden for patients. Novel CAR-T cell designs can potentially improve antigen recognition on lymphoma cells and enhance CAR-T cell persistence while minimizing the risk of CRS and ICANS. For patients with a high disease burden requiring urgent therapy, exploring off-the-shelf allo-CAR cells, which do not require individualized manufacturing and can be cryopreserved and banked in batches, may help overcome logistical and financial challenges. This approach has the potential to reduce lead times and create a more accessible platform for cell therapies.^110^

## Nanotechnology perspectives

 Integrating CAR-T cells with nanotechnology has emerged as a promising approach for lymphoma treatment. Nanotechnology offers unique advantages in enhancing CAR-T cells’ efficacy, specificity, and delivery, addressing some of the limitations associated with conventional CAR-T cell therapy.

 One critical application of nanotechnology in CAR-T cell therapy is the design and development of nanoparticles for the targeted delivery of CAR-T cells to the tumor site. Engineered nanoparticles can carry CAR-T cells and selectively target tumor cells, thereby improving therapeutic outcomes. Functionalizing nanoparticles with specific ligands or antibodies allows for precise recognition and binding to tumor-associated antigens, facilitating the targeted delivery of CAR-T cells to lymphoma cells while minimizing off-target effects.

 Furthermore, nanotechnology provides opportunities to enhance the functionality of CAR-T cells. Surface modification of CAR-T cells with nanomaterials can improve their persistence and cytotoxicity against lymphoma cells. For instance, integrating nanomaterials such as mesoporous silica nanoparticles or graphene oxide with CAR-T cells can enhance their survival, expansion, and tumor-killing abilities. These nanomaterials can also serve as carriers for immunomodulatory drugs, cytokines, or gene editing tools, enabling the on-demand release of therapeutic agents to further enhance the anti-tumor response of CAR-T cells.

 Nanotechnology also enables precise control over the spatiotemporal release of immunomodulatory factors to modulate the TME and overcome immunosuppression. Nanoparticles loaded with immune checkpoint inhibitors, such as PD-1 or CTLA-4 inhibitors, can enhance the activation and function of CAR-T cells within the TME. Moreover, nanoscale drug delivery systems can be engineered to release immunomodulatory agents in a controlled manner, thereby improving the overall therapeutic efficacy of CAR-T cell therapy for lymphoma.

 In addition to improving the delivery and functionality of CAR-T cells, nanotechnology offers opportunities for real-time monitoring and imaging of CAR-T cell biodistribution and tumor response. By incorporating imaging agents, such as fluorescent dyes or nanoparticles, CAR-T cells can be visualized in vivo, assessing their migration, homing, and persistence in lymphoma lesions. This real-time monitoring enables clinicians to evaluate the effectiveness of CAR-T cell therapy and make informed decisions regarding treatment strategies.

 Overall, incorporating nanotechnology into CAR-T cell therapy holds great potential for enhancing lymphoma treatment. Leveraging the unique properties of nanoparticles and nanomaterials can improve CAR-T cell delivery, functionality, and monitoring, ultimately leading to more effective and targeted therapies for lymphoma patients. Continued research and development in this interdisciplinary field will pave the way for translating nanotechnology-based CAR-T cell therapies into clinical practice, offering new avenues for personalized and precision medicine in lymphoma treatment.

## Bioinformatics and AI perspectives

 Artificial intelligence (AI) and machine learning (ML) are revolutionizing CAR-T cell therapy for lymphoma and beyond. From identifying ideal patients and navigating potential complications to optimizing CAR design and streamlining production, these powerful tools are transforming every facet of this life-changing treatment. AI algorithms predict responses, personalize therapy, unveil novel targets, and automate manufacturing, paving the way for a future where CAR-T conquers more cancers, saving more lives.^111^

 The fusion of bioinformatics and AI has revolutionized the development and design of CARs. Bioinformatics, which combines computer science, statistics, and biology, is instrumental in analyzing biological data and identifying optimal scFv sequences for CAR construction. AI, a branch of computer science dedicated to intelligent machines, complements bioinformatics by devising algorithms that analyze data and predict the most effective CAR combinations. AI technologies profound learning, have significantly improved the accuracy and precision of CAR design. Deep learning algorithms discern patterns within data, facilitating the creation of CARs with highly efficient scFv combinations. This integration empowers scientists to craft CARs with heightened specificity, improved affinity, and enhanced efficacy in targeting specific antigens.

 In the context of CAR-T cell therapy, AI plays a crucial role in several aspects, including predictive modeling, personalized medicine, and target identification. AI algorithms, such as ML, enable the creation of predictive models that can anticipate patient responses to CAR-T cell therapy. By analyzing extensive datasets of patient characteristics, genetic profiles, and treatment outcomes, AI can identify patterns and factors that influence therapy effectiveness. AI allows for the customization of CAR-T cell therapies based on individual patient profiles. This involves tailoring the design of CAR constructs to target specific antigens present on the patient’s cancer cells. AI-driven approaches help optimize the therapeutic potential of CAR-T cells for each patient. AI algorithms can analyze vast biological databases to identify novel target antigens for CAR-T cell therapies. By processing genomic and proteomic data, AI aids in discovering unique markers on cancer cells that can be targeted by CAR-T cells, expanding the scope of potential therapies.

 Current databases are pivotal in optimizing CAR design by offering various scFv sequences. Analyzing these databases assists scientists in identifying the most suitable scFv sequences to integrate into other CAR constructs, further refining CAR design.

 The synergy of bioinformatics and AI has led to significant advances in CAR design, such as the identification of new target antigens and the creation of more effective CARs for killing cancer cells. This powerful combination has the potential to lead to the development of more precise and personalized CAR therapies for cancer.^111^

 Therefore, bioinformatics, AI, and current databases are indispensable tools for developing optimal CARs. The amalgamation of bioinformatics, AI, and ML aids in pinpointing suitable scFv sequences for CAR construction. Additionally, current databases provide valuable insights for fine-tuning CAR design. By harnessing these tools, scientists can create CARs with highly efficient and precise scFv combinations.

## Machine Learning Perspective

 The emergence of CAR T-cell therapy has provided a promising avenue for the treatment of lymphoma, a group of blood cancers that has traditionally been challenging to address. This innovative approach involves genetically engineering a patient’s own T cells to target and eliminate cancer cells, resulting in notable remission rates. To further enhance the efficacy and personalization of CAR-T therapy for lymphoma, Artificial Intelligence (AI) and Machine Learning (ML) play a pivotal role in several key areas.

 AI and ML are instrumental in identifying optimal target antigens on lymphoma cells, which is crucial for CAR-T design. By analyzing extensive genomic and proteomic data from tumor samples, these technologies can uncover novel target antigens that may not be readily apparent to human researchers, potentially leading to the development of more effective CAR-T constructs.^112^

 AI and ML contribute to the optimization of CAR design by predicting the most effective combination of signaling domains, T cell costimulatory molecules, and spacer sequences within the CAR construct. This data-driven approach holds the promise of creating more potent and less prone to side effects CAR-T cells.^113^

 AI and ML help predict patient response to CAR-T therapy by analyzing a patient’s medical history, genetic profile, and tumor characteristics. This personalized prediction enables clinicians to tailor treatment options and manage expectations, thereby enhancing the overall therapeutic strategy.

 The integration of AI and ML in CAR-T therapy represents a significant advancement in the fight against lymphoma. By streamlining design, enhancing efficacy, and personalizing treatment, these technologies offer a new dawn in the battle against this devastating disease, potentially transforming once hopeless diagnoses into hopeful outcomes.^112^

 Over the years, ML has become crucial for immunotherapeutic applications. A prominent application is peptide binding affinity prediction, which identifies targets for TCR-T treatment or customized neoantigen vaccination. The accuracy of binding affinity estimates has improved significantly, thanks to ongoing developments in ML algorithms and the availability of training data. While CD8 + cytotoxic T cells have received substantial attention, CD4 + T cells also play a significant role. Consequently, efforts are underway to enhance predictions for MHC class II presented epitopes, which is more challenging due to the broader range of peptide lengths and the open binding groove.^114^

 Despite the progress in ML, there are still challenges, particularly in understanding immunogenicity, especially in personalized therapy based on neoepitope identification. This complexity arises from the unique and heterogeneous interactions between a patient’s immune system and tumor. To advance more reliable personalized immunotherapy, researchers are focusing on identifying immune system features that influence T cell recognition of individual epitopes in tumors, thereby benefiting multiple patients.^115^

 Immunotherapy offers the advantage of fewer side effects compared to existing cancer treatments. Enhanced bioinformatics tools and prediction algorithms have the potential to make immunotherapy more precise, customized, and effective.

 However, several obstacles must be overcome to increase the use of CAR-T cells in standard clinical practice. These challenges include addressing technical difficulties in CAR-T cell development and manufacturing, standardizing clinical trial results for meaningful comparisons (including protocols, patient pre-conditioning, CAR-T cell formulation, quality, and persistence), and identifying reliable tools to optimize treatment decisions.^116,117^

 Researchers have developed an optimized process for designing specific scFvs with strong binding affinities. This process uses ML techniques to integrate target-specific binding affinities and information from protein sequences. The approach involves high-throughput binding quantification, pre-training language models, fine-tuning models for binding affinity prediction, constructing a fitness landscape, in silico scFv design using Bayesian optimization, and experimental validation. Training data is generated using a yeast mating assay, and language models are trained for affinity prediction. A Bayesian-based fitness landscape is constructed, and sampling algorithms are employed to generate high-affinity scFv libraries. These libraries are experimentally tested, and the top sequences are selected for further analysis.

 In the context of CAR-T cell therapy and bioinformatics, ML plays a crucial role in several aspects, including feature selection, clustering and classification, pattern recognition, and automated decision support.

 Feature Selection: ML techniques are employed to identify the most relevant features or variables from complex biological data. This helps in selecting the key factors that influence treatment outcomes and guide the development of CAR-T cell therapies. ML algorithms can categorize patients into distinct groups based on their genetic profiles and disease characteristics. This enables the identification of patient subpopulations that may respond differently to CAR-T cell therapy, allowing for tailored treatment strategies. ML models excel at recognizing subtle patterns and associations within large-scale genomic and proteomic datasets. This capability aids in uncovering potential biomarkers, predicting treatment responses, and optimizing CAR-T cell design. Automated Decision Support: ML-based decision support systems can assist healthcare professionals in making informed treatment decisions by providing real-time analysis of patient data and treatment options

## Convolutional neural network (CNN)

 Computer vision (CV) has gained traction in autonomous technology and advanced surveillance systems. In recent years, there has been a surge in research on medical image processing to aid physicians in disease diagnosis and identify features that may go unnoticed. Unlike doctors, CV can work continuously and efficiently while maintaining decent accuracy. The conventional approach involves ML, where pre-processing techniques like principal component analysis, independent component analysis, and linear discriminant analysis extract key information. However, this method may miss important features, leading to lower accuracy. Classification methods can be categorized as supervised learning (e.g., support vector machine, k-nearest neighbor, classification and regression tree) and unsupervised learning (e.g., K-Means). ML requires sufficient information to achieve higher accuracy, prompting the exploration of alternative methods to overcome this challenge.

 With the advancement of computer technology, new hardware and software have emerged that offer larger memory capacity and higher processing power for deep learning (DL) training. DL overcomes the limitations of traditional ML. CNNs play a crucial role in DL, comprising multiple convolutional layers with kernels of varying sizes, enabling end-to-end learning. CNN utilizes these convolutional kernels to extract a multitude of features and combine them to accomplish downstream tasks. The application of CNN in medical image processing has garnered interest from both researchers and doctors. When it comes to cell classification, outer phenotype and inner structure are commonly used input features. Cell recognition reports encompass various cell types, including blood cells, cancer cells, and others.

 Several models have been developed and adapted for cell recognition. One effective approach involves a hybrid model that combines transfer learning and generative adversarial networks (GANs) to enhance the accuracy of a small dataset containing staining-free cancer cells. This model utilizes optical path delay maps obtained from low-coherence off-axis holography as input and pretrains the GANs with sperm cells before training the main dataset. The accuracy achieved by this model is 99%, surpassing the performance of single GANs or MobileNet with transfer learning.^112^

 In another study, an accuracy of 99.54% is achieved by combining DL and support vector machine for classifying sickle cells and normal blood cells. This approach incorporates transfer learning and data enhancement techniques. Moreover, a deeply supervised residual network demonstrates an accuracy of 99.98% in classifying human epithelial-2 cells.

 For the analysis of cellular inner structure, DL proves effective in classifying Cercopithecus aethiops monkey kidney cells based on microtubule networks, outperforming human experts. Additionally, CNN shows promise in distinguishing normal and cancer cells in breast tissue by identifying discrepancies in actin cytoskeleton structures, serving as an additional diagnostic marker with superior performance compared to human experts.

 CNNs possess strong inductive biases, such as local correlation and weight sharing, which enhance accuracy and effectiveness. However, CNN also imposes limitations on the upper performance bound of the model. While deeper CNNs can mitigate the impact of limited receptive fields and long-range dependencies, they require more complex and larger architectures, making training more challenging. On the other hand, the Transformer model excels in capturing global correlations and has been widely successful in natural language processing. However, Transformer training is time-consuming and demands an extensive dataset and high computational memory.

 Efforts have been made to combine CNN and Transformer to leverage their respective strengths and achieve improved results. For example, the ViT model crops multiple patches from an image and reshapes them to resemble word embeddings, thereby employing the Transformer structure with modified input. Another hybrid model, AA-ResNet, integrates CNN and Transformer and achieves an accuracy of 77.7% on ImageNet classification. However, a comprehensive Transformer is intricate and less adaptable for transferring from text processing to image processing. The Bottleneck Transformer (Bot) block addresses this by using the core self-attention mechanism from Transformers to replace the middle convolutional layer in ResNet50’s last blocks. Although the model structure remains largely unchanged, it attains higher accuracy compared to traditional CNN approaches.

## CAR-T DATASET

 To the best of our knowledge, no existing CAR-T cell dataset is currently available. Therefore, in this work, we have constructed the first CAR-T cell dataset, which can serve as a baseline and reference for future research and dataset construction in this field.

 The CAR-T cell dataset was obtained from six patients with acute lymphoblastic leukemia who received CAR-T therapy at the First Affiliated Hospital of Harbin Medical University. Blood samples were collected from these patients within several days or weeks after CAR-T therapy. All patients provided informed consent for the study, and the protocol received ethical approval from the hospital’s ethical committee. The blood samples were stained using the May Grünwald-Giemsa method, and CAR-T cells were confirmed through immunostaining for CAR-T specific markers. Wide-field microscopy with a 100x oil immersion lens was used to capture CAR-T cell images, with each image having a size of 384 × 384 pixels and containing only one cell.

 To label the CAR-T cells in the dataset, we collaborated with professional blood morphologists due to the complexity and scarcity of blood samples. The dataset consists of two categories of cells: CAR-T cells and other normal cells. We collected 250 images per class, with an assignment ratio of 8:2 for each class.^113^

## AI in CAR-T

 Therapy involving CAR T-cells is a technologically advanced cancer treatment method based on adoptive cellular immunotherapy. It uses the patient’s own T-cells, which are genetically modified outside the body to express the CAR receptor specific to the tumor antigen. These reprogrammed T lymphocytes are then administered intravenously, where they expand, recognize, and eliminate cancer cells. CAR T-cell therapy has shown great effectiveness in treating B-cell lymphomas, B-cell acute lymphoblastic leukemia, and multiple myeloma. It has the potential to complement conventional treatment and hematopoietic stem cell transplantation. Ongoing clinical trials suggest its efficacy in early disease phases. However, the treatment is associated with unique toxicities that may limit its use. Additionally, CAR T-cell therapy is expensive and requires advanced technology for production. Precise qualification, monitoring, and possible interventions are necessary. Artificial Intelligence can be used to combine biomarkers associated with CAR T-cell response to create robust prognostic/predictive models. Building such models using AI requires large datasets, necessitating data aggregation from multiple institutions to avoid overfitting.^113^

## Future manufacturing perspective

 This perspective explores early ideas regarding the potential application of automated AI-driven CAR-T cell production directly at the point of care within hospitals, emphasizing engineering considerations for making hardware and software components efficient in manufacturing autologous CAR-T cells. Significant barriers must be addressed before widespread deployment in the regulatory landscape. While facility design according to GMP standards and Government-Mediated Access Price (GAMP) recommendations have been contemplated for software, specific regulatory guidance from the EMA and FDA is essential before implementing an AI-driven manufacturing platform. It involves ensuring the AI algorithm’s reliability and ability to operate confidently, including aspects such as the quality and quantity of data suitable for training and potentially for continuous training.^118,119^

 Economic factors are also a key consideration. The reduction in labor costs compared to automation costs is a critical aspect. Demonstrating the potential for overall cost reduction is achievable by comparing similar technologies for automated stem cell production.^120^ In future developments of AI-driven Decentralized Production for Advanced Therapies in the Hospital (AIDPATH), economic health assessments will be a focus, considering patient supply considerations alongside economic factors. Due to parallelization, bioreactors can strive for high scalability and throughput, which can increase patient access through reduced production and delivery timelines. The extent to which centralized and decentralized CAR-T cell production coexists in the future requires further discussion, as does the operator model.

 While not everyone needs highly specialized skills, the system can be operated by individuals with the flow working automatically, featuring an easy-to-use user interface. The role of AI in hospitals, external service providers, or pharmaceutical companies remains uncertain, and it is essential to consider that maintenance and operation of hardware, AI, and IT infrastructure will necessitate new responsibilities for operators.^121^

 In summary, this concept presents a promising idea that will require ongoing refinement and expansion in the coming years to keep pace with the rapidly evolving cell and gene therapy market.^122^

 To further enhance the understanding of this concept, it is essential to consider the interdisciplinary exchange of stakeholders and the development of a holistic approach to exploit the full potential of automated CAR-T cell therapy. The deployment of an automated AI-driven CAR-T cell manufacturing platform in smart manufacturing hospitals is a significant step toward this future. The concept, developed within the scope of EU H2020 project AIDPATH (AI-driven, Decentralized Production for Advanced Therapies in the Hospital), focuses on addressing technological challenges and potential solutions, emphasizing the need for regulatory guidance and economic health assessments.

 The visual representation of an automated CAR-T cell production system within a hospital setting, along with data flow diagrams and scalable bioreactors, can provide a vivid and comprehensive understanding of the potential of this technology. These visuals can help stakeholders grasp the intricacies of the AI-driven process and the potential for increased patient access through faster production times

## Future medical imaging perspective

 Among these evolving technologies, medical imaging methods hold significant importance. The utilization and purposes of medical imaging are still evolving, but the findings presented above strongly indicate that imaging will play a pivotal role in guiding treatment decisions. Recent research suggests that in a cohort of patients with DLBCL who received first-line chemotherapy, the assessment of lesion dissemination on PET/CT, measured by the most significant distance between two lesions (normalized with body surface area), contributes to evaluating disease spread and holds independent prognostic value, regardless of Total Metabolic Tumor Volume.^123^

 Radiomics, another emerging field, can assist in extracting clinically relevant information from medical imaging. Recent studies across various solid tumor types suggest that a signature composed of a small subset of imaging biomarkers, whether assessed before^124^ or during^124,125^ treatment, may help identify individuals who could benefit from early intervention.^126^ Several quantitative imaging biomarkers, including increased tumor volume, higher tumor glucose metabolism, tumor organotropism in visceral organs, and reduced skeletal muscle index, have demonstrated the potential to predict responses to immunotherapies involving immune checkpoint blockers. Importantly, all of these biomarkers exhibit a negative correlation with treatment outcomes.^127–129^

## Cost analysis in the future

 In the current stage of development, researchers are still assessing the cost-effectiveness of CAR-T treatment while considering the inherent risks associated with large-scale CAR-T production. An in-depth analysis of CAR-T cell therapy necessitates labor-intensive laboratory tests conducted by experienced professionals. Most of these processes must adhere to specific GMP standards to ensure the success of CAR-T production. Compared to other cancer treatment methods, CAR-T treatment involves several stages that come with higher costs, including virus-based gene delivery, the extensive use of cell line cultures, and CAR-T safety testing for each recipient.^130,131^

 Efforts to reduce the costs of CAR-T production involve optimizing various stages. It includes selecting the most efficient vector for gene delivery, researching viruses with the highest competence in transducing target genes, employing optimal techniques for cell packaging lines in cGMP production, and minimizing additional post-infusion care required for CAR-T-treated patients.^130^

 In contrast, some patients have compared the costs of CAR-T treatment infusion to traditional chemotherapy, considering factors like the stage of cancer, the potential side effects of chemotherapy, the costs associated with post-infusion CAR-T care, and the more promising outcomes.^132,133^ While selecting CAR-T patients based on specific criteria has been implemented, it has not always yielded optimal results—additionally, patients who do not exhibit a treatment response after CAR-T treatment risk incurring treatment costs. Therefore, extensive testing before CAR-T infusion treatment is necessary to minimize unnecessary expenses.^134^

 Governments have expressed concerns about CAR-T treatment due to its high cost and the need for complex legal permits. However, scientific advancements deserve recognition, even if all do not readily accept them. As a result, various efforts are needed to enhance knowledge, streamline regulatory logistics, and allocate funds for further development. Current technology strongly supports the evolution of CAR-T treatment, thanks to contributions from scientists aiming to make CAR-T treatment more efficient and accessible. These considerations are crucial, as preliminary studies have shown the effectiveness of CAR-T treatment in saving cancer patients.^135^

## Conclusion

 Lymphoma, one of the cancer types requiring increased attention in its treatment, still faces limitations in available medications on a global scale. As an immunotherapy tool, CAR-T cell therapy holds the promise of a significant breakthrough in the treatment of lymphoma cancer. To gain a comprehensive understanding of cancer treatment using CAR-T cell therapy technology, one must delve into detailed information and consider which types of cancer treatments should be explored and developed in future manufacturing.

 This review has focused on the evolution of CAR-T cell therapy over the years, the selection of appropriate lymphoma antigens for CAR-T targeting, and the design, production, and administration of CAR-T cell therapy. It has also presented the advantages and drawbacks of this therapy, supplemented by insights into the future potential of CAR-T cell therapy from a computational study perspective. This information is anticipated to provide valuable insights for researchers and other stakeholders in developing this promising technology for more effective lymphoma cancer treatment.

## Acknowledgments

 We thank Nur Rahmah Awaliah for her editing services and technical support.

## Competing Interests

 None.

## Ethical Approval

 Not applicable.
